# Efficacy and Safety of Supramolecular Salicylic Acid Combined With Intense Pulsed Light for Rosacea: A Split‐Face Trial

**DOI:** 10.1111/jocd.71007

**Published:** 2026-06-22

**Authors:** Meifang Wang, Ansheng Tan, Fenglin Zhuo

**Affiliations:** ^1^ Department of Dermatology Beijing Friendship Hospital, Capital Medical University Beijing China

**Keywords:** combined therapy, intense pulsed light (IPL), rosacea, split‐face study, supramolecular salicylic acid (SSA)

## Abstract

**Background:**

Rosacea is a chronic inflammatory skin disorder with a global prevalence of approximately 5.46%, significantly impairing patients' quality of life and mental health. We aimed to assess the efficacy and safety of supramolecular salicylic acid (SSA) peeling combined with intense pulsed light (IPL) for rosacea and its impact on skin barrier function.

**Methods:**

Thirty‐six patients with erythematotelangiectatic or papulopustular rosacea were enrolled in this split‐face study. All patients received full‐face IPL treatment monthly for three sessions; a randomly assigned facial half underwent a 30% SSA peel per session. Efficacy was assessed using the VISIA Complexion Analysis System and the CK multiple probe adapter. The Clinical Erythema Assessment (CEA) and patient self‐assessment served as secondary outcomes. Skin hydration and transepidermal water loss (TEWL) were monitored to evaluate the skin barrier.

**Results:**

Thirty‐three patients completed the trial (three dropouts). After 3 months, the combination side showed significantly greater reductions in VISIA erythema scores (−3.32 ± 3.81 vs. −1.88 ± 2.58; *p* = 0.0041) and CK erythema index (−36.09 ± 20.80 vs. −25.88 ± 25.86; *p* = 0.0013) than the IPL‐alone side. Both sides showed significant improvement in CEA and patient self‐assessment (*p* < 0.05), with no statistically significant inter‐side difference. Skin hydration increased while TEWL decreased bilaterally (*p* < 0.05). Adverse events were mild and comparable between sides (*p* = 0.476).

**Conclusion:**

SSA peeling combined with IPL is superior to IPL monotherapy for rosacea erythema, offering enhanced efficacy without compromising skin barrier function.

## 1 Introduction

Rosacea is a chronic inflammatory skin disorder with a global prevalence of approximately 5.46%. The erythematotelangiectatic (Type I) and papulopustular (Type II) subtypes are the most common clinical subtypes [1, 2]. Typical symptoms include paroxysmal flushing, persistent erythema, inflammatory papules, and pustules, often accompanied by sensations such as burning, stinging, dryness, or edema [3], which severely impact patients' mental health and quality of life [4]. Current treatment modalities include topical medications, systemic therapy, and phototherapy, each of which has its limitations [5].

As a common photoelectric treatment, intense pulsed light (IPL) exerts its effect by emitting light that is selectively absorbed by hemoglobin, generating thermal energy that can improve erythema and telangiectasia symptoms in rosacea [6]. However, its efficacy is limited regarding active inflammatory papules, sebaceous gland hyperplasia, or individuals with dark skin phototypes, and it is more suitable for patients in the stable phase [7].

Supramolecular salicylic acid (SSA) is based on supramolecular self‐assembly technology, in which salicylic acid is combined with a poloxamer carrier via reversible non‐covalent bonds to form a stable hydrogel complex system. This allows SSA to exert its pharmacological effects effectively while greatly reducing irritation to the skin barrier. Currently, some studies have confirmed that 30% salicylic acid has good efficacy in improving the symptoms of rosacea [8, 9]. The regulatory effects of SSA on the skin barrier potentially complement the mechanism of IPL, which targets dermal vasculature and tissue, suggesting a potential synergistic effect for combination therapy. However, there is still a lack of high‐quality research with prospective, randomized, self‐controlled designs to evaluate the efficacy of IPL combined with SSA.

Therefore, this study aims to systematically evaluate the effectiveness and safety of the SSA combined with IPL for rosacea via a comprehensive assessment using the VISIA Complexion Analysis System, the CK multiprobe adapter system, Clinician's Erythema Assessment (CEA), and patient self‐assessment of symptoms, to provide robust evidence for clinical management of this disease.

## 2 Methods

This was a prospective, randomized, split‐face, self‐controlled clinical trial. The study protocol was approved by the Ethics Review Committee of a tertiary hospital. All participants provided their signed informed consent. The study was conducted in accordance with the Declaration of Helsinki of 1964 and its later amendments.

### 2.1 Study Population

Eligible participants were patients aged 18–55 years diagnosed with erythematotelangiectatic (Type I) or papulopustular (Type II) rosacea. Exclusion criteria included: Concurrent facial dermatoses that could interfere with efficacy assessment; a known allergy to salicylic acid or a history of photosensitivity disorders; use of topical medications, chemical peels, or photodynamic therapy within the preceding two weeks; systemic treatment for rosacea or acne (including steroids, antibiotics, and isotretinoin) within the preceding four weeks; diagnosis of malignant tumors or other severe organic or psychiatric diseases; and pregnancy or lactation. The researchers assessed the eligibility of all potential subjects through face‐to‐face interviews.

### 2.2 Randomization

A computer‐generated random number table was used for allocation. Eligible patients were assigned to receive the combination therapy (IPL + SSA) on the left side and IPL‐alone on the right side if they were assigned an odd number; this assignment was reversed for patients with even numbers.

### 2.3 Study Design and Procedure

The total study period was 12 weeks. All patients received treatments at weeks 0, 4, and 8, with follow‐up assessments conducted at weeks 0 (T0), 4 (T1), 8 (T2), and 12 (T3).

Before each treatment session, patients underwent facial cleansing and rested in a designated environment for 20 min. At the beginning of the treatment, a 30% SSA preparation was applied exclusively to the combination side for 10 min, followed by rinsing with water. A cold compress incorporating a hyaluronic acid mask was applied for 20 min, accompanied by a dynamic cooling system spray. The SSA application was discontinued, and the agent was rinsed off immediately if the patient experienced significant discomfort or frosting. Subsequently, IPL treatment was administered to the entire face. The parameters were set as follows: Wavelength 560/590 nm, triple‐pulse mode, fluence 12–16 J/cm^2^. The treatment endpoint was defined as mild erythema or transient purpura. The same cold compress and cooling spray procedure was repeated after the IPL treatment.

For post‐procedure care, all patients were instructed to use the provided soothing and reparative essence and cream daily and to practice strict sun protection. They were also required to refrain from using any other functional skincare products for the duration of the study.

### 2.4 Evaluation and Outcome Measures

#### 2.4.1 Facial Image Analysis

Standardized facial images of patients were captured at each follow‐up time point using the VISIA Complexion Analysis System. Quantitative analysis of “Red Areas” (erythema), texture, and pores was performed.

#### 2.4.2 Skin Physiological Function Testing

Skin physiological parameters, including the erythema index (EI), epidermal hydration, and transepidermal water loss (TEWL), were measured using the CK multiple probe adapter. During measurement, the probe was gently pressed vertically against the skin surface at the predefined site (the midpoint of the line connecting the nasal root and the preauricular area). Each parameter was measured three times consecutively, and the average value was calculated.

#### 2.4.3 Clinical Erythema Assessment (CEA)

The CEA scale was employed by two independent clinicians to score erythema severity: 0, clear skin with no signs of erythema; 1, slight redness (almost clear); 2, definite redness (mild erythema); 3, moderate erythema (marked redness); 4, severe erythema (fiery redness).

#### 2.4.4 Patient Self‐Assessment of Symptoms

Patients rated the severity of three symptoms—facial redness, itching, and burning sensation—using a four‐point Likert scale: 0, none; 1, mild; 2, moderate; 3, severe. Based on the changes in these scores, the degree of improvement was categorized as follows: Grade I (“Minimal”), no change in score; Grade II (“Moderate”), 1‐point reduction; Grade III (“Marked”), 2‐point reduction; Grade IV (“Excellent”), 3‐point reduction.

#### 2.4.5 Dermoscopy

Dermoscopic images of the bilateral cheek and nasal regions were captured to observe and compare changes in microvascular morphology and skin microstructure between the combination side and the IPL side.

#### 2.4.6 Safety Evaluation

At each treatment and follow‐up visit, the same investigator recorded any treatment‐related adverse events, such as pain, pruritus, burning sensation, as well as erythema, dryness, desquamation, blisters, erosion, crusting, and hyperpigmentation. The severity, duration, and outcome of all adverse events were documented.

#### 2.4.7 Satisfaction

At Week 12, patients rated their overall satisfaction using a five‐level scale: Very dissatisfied, dissatisfied, neutral, satisfied, and very satisfied. The overall satisfaction rate was calculated as follows: [(satisfied + very satisfied)/total patients] × 100%.

#### 2.4.8 Sample Size

The sample size was determined based on preliminary data from our pilot study (*n* = 10). Using PASS software (version 15.0) and a paired‐design model, we calculated that a minimum of 33 participants was required to detect a significant difference in the improvement of the VISIA erythema score, with a statistical power (1‐β) of 80% and a significance level (α = 0.05). Accounting for a predicted 10% dropout rate, we aimed to enroll a total of 36 patients.

#### 2.4.9 Statistical Analysis

Statistical analysis was performed using SAS software (version 9.4). Normally distributed continuous data are presented as mean ± standard deviation (SD), while non‐normally distributed continuous data are presented as median (interquartile range). Categorical and ordinal data are expressed as a number (percentage). The paired *t*‐test was used for continuous variables for the split‐face comparisons. All tests were two‐sided, and *p* < 0.05 was considered statistically significant. For patients who dropped out, the primary efficacy analysis used the last observation carried forward (LOCF) method to impute missing data, replacing post‐treatment missing values with the valid data from the patient's most recent visit. To test the robustness of the primary analysis conclusions, we also performed a sensitivity analysis.

## 3 Results

### 3.1 Demographic Characteristics (Table 1)

**TABLE 1:** Demographics of the enrolled study patients.

Characteristics	*n* (%)
Completed study	33/36 (91.7)
Dropped out	3/36 (8.3)
Sex^a^	
Male	4/33 (12.1)
Female	29/33 (87.9)
Rosacea type^a^	
I	23/33 (69.7)
II	10/33 (30.3)
Fitzpatrick skin type^a^	
III	18/33 (54.5)
IV	15/33 (45.5)
Age^a^	
18–30	5/33 (15.2)
31–40	12/33 (36.4)
41–50	14/33 (42.4)
51–55	2/33 (6.1)

^a^ Includes patients who completed the study (*n* = 33).

A total of 36 patients were enrolled in this study. Thirty‐three patients (91.67%) completed all follow‐ups, while three (8.33%) withdrew: One patient withdrew due to intolerance of aggravated transient inflammatory reaction after the first treatment (recorded as an adverse event); another patient withdrew for personal time reasons; and one patient was lost to follow‐up. These 33 completers comprised 29 females and 4 males. According to the Fitzpatrick skin phototype classification, 18 patients were type III and 15 were type IV.

### 3.2 Primary Outcome (LOCF)

#### 3.2.1 VISIA Image Analysis

**FIGURE 1:**
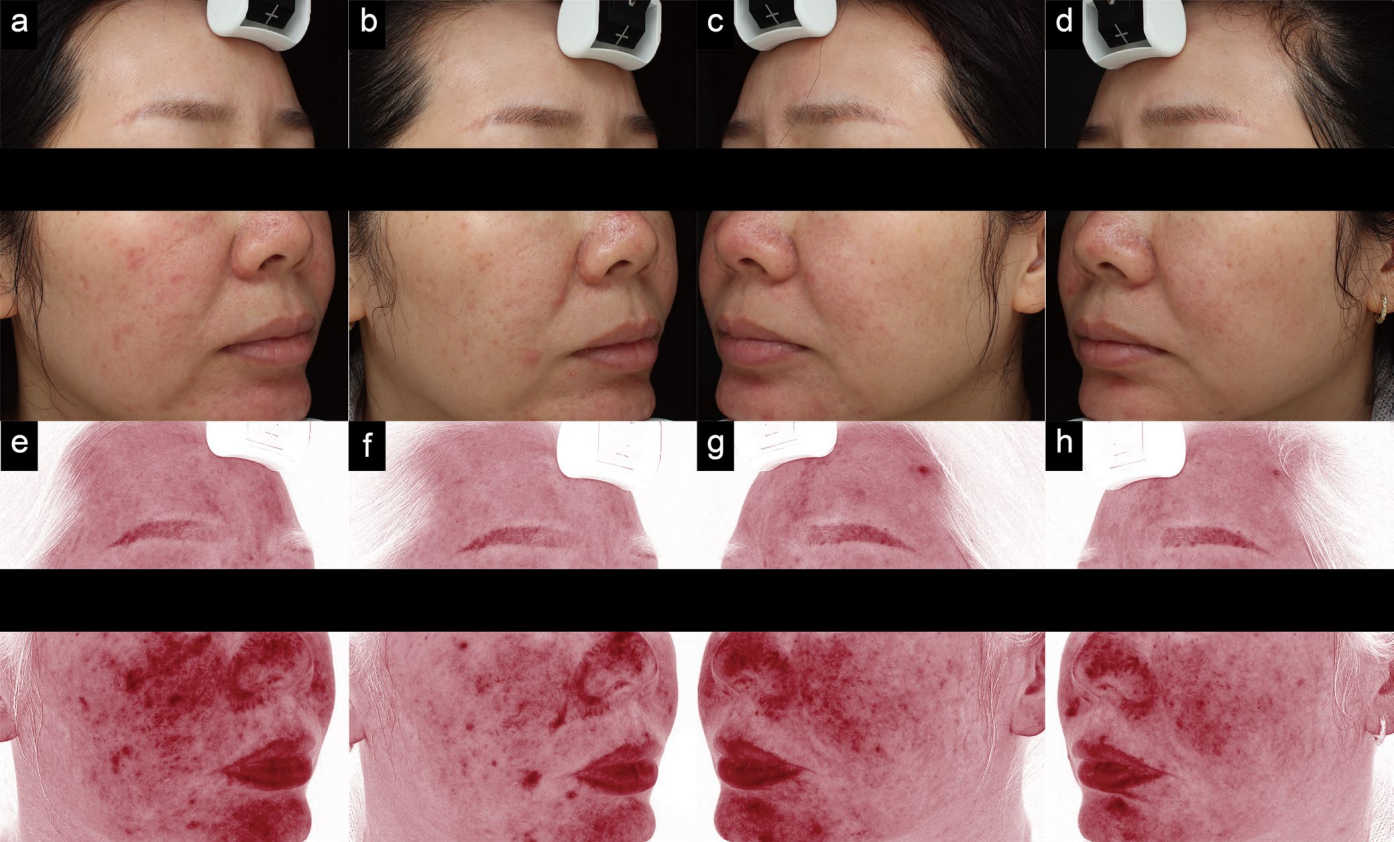
Comparison of VISIA images at baseline and the final follow‐up (Week 12). (a–d) Standard facial photographs of a representative patient with rosacea: Baseline (a, combination side; c, IPL‐alone side) and Week 12 (b, combination side; d, IPL‐alone side). (e–h) Corresponding VISIA “Red Area” analysis images: Baseline (e, combination side; g, IPL‐alone side) and Week 12 (f, combination side; h, IPL‐alone side).

**FIGURE 2:**
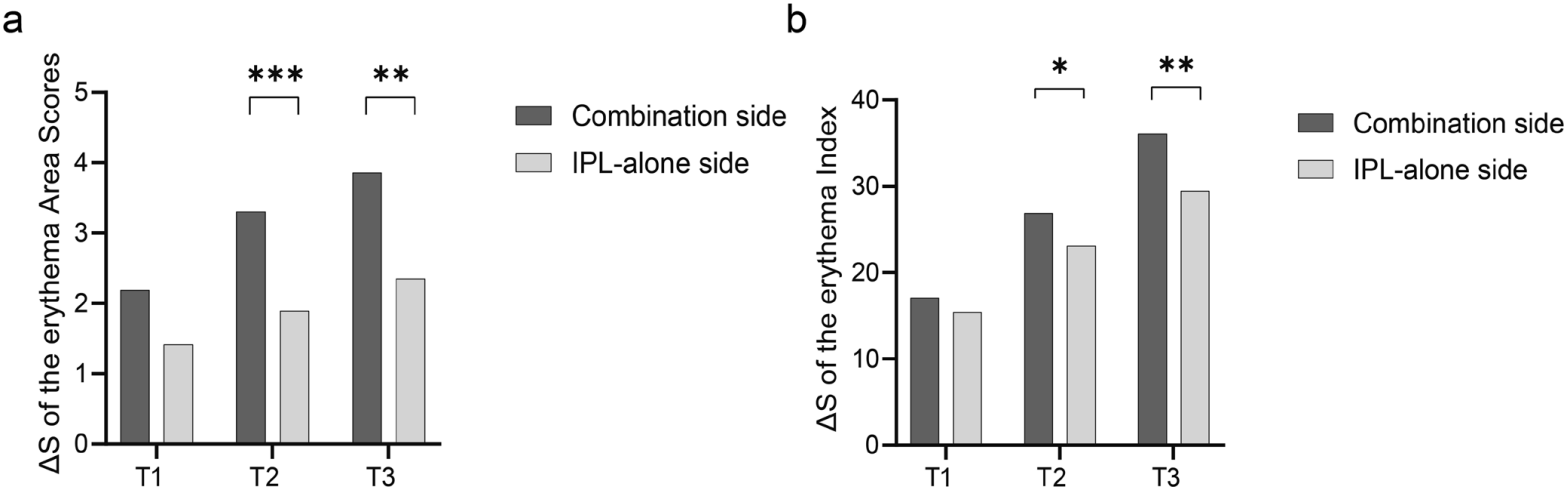
Changes in erythema parameters from baseline across follow‐up visits. (a) Reduction in VISIA erythema area scores. The combination side showed a significantly greater reduction than the IPL‐alone side at T2 and T3 (*p* < 0.001 and *p* = 0.0041, respectively). (b) Reduction in the CK erythema index. The combination side showed a significantly greater reduction than the IPL‐alone side at T2 and T3 (*p* = 0.0298 and *p* = 0.0013, respectively).

The Red Areas for both the combination and IPL‐alone sides exhibited a decreasing trend as the treatment progressed. The reduction in erythema area score (ΔS = Tn—T0) did not differ significantly between sides after the first treatment (−1.81 ± 2.96 vs. −0.95 ± 1.85; *p* = 0.072). However, after the third treatment, the combination side showed a significantly greater reduction than the IPL‐alone side (−3.32 ± 3.81 vs. −1.88 ± 2.58; *p* = 0.0041; Figure 1a–h; Figure 2a).

A similar improvement trend was observed for pore and texture scores, which decreased progressively on both sides. For pore scores, the reduction was significantly greater on the combination side than on the IPL‐alone side at both T2 (*p* = 0.005) and T3 (*p* < 0.001). For texture scores, a significantly greater reduction was already evident on the combination side after the first treatment (T1: *p* = 0.020) (Table 2).

**TABLE 2:** Objective assessment of skin parameters at baseline and post‐treatment (mean ± SD).

	Time	Combination Side	ΔS = Tn‐T0	IPL‐alone side	ΔS = Tn‐T0	*p*‐value (ΔS)
**VISIA Parameters** ^a^						
**Red Areas**	T0	42.85 ± 7.88		41.27 ± 7.72		
	T1	40.41 ± 8.35	−1.81 ± 2.96	40.17 ± 8.06	−0.95 ± 1.85	0.0720
	T2	38.86 ± 8.29	−3.36 ± 3.74	39.38 ± 8.64	−1.73 ± 2.47	0.0001
	T3	39.52 ± 8.95	−3.32 ± 3.81	39.39 ± 7.66	−1.88 ± 2.58	0.0041
**Pores**	T0	19.78 ± 8.14		20.01 ± 7.90		
	T1	19.07 ± 8.07	−1.30 ± 1.58	19.58 ± 8.25	−0.93 ± 1.14	0.1537
	T2	18.12 ± 7.82	−2.25 ± 1.80	19.05 ± 7.87	−1.47 ± 1.14	0.0057
	T3	17.51 ± 7.96	−2.86 ± 1.88	18.66 ± 7.91	−1.86 ± 1.71	0.0001
**Texture**	T0	7.76 ± 3.34		7.17 ± 2.99		
	T1	7.13 ± 3.32	−0.89 ± 0.77	6.86 ± 2.98	−0.53 ± 0.78	0.0211
	T2	6.47 ± 2.90	−1.56 ± 1.00	6.40 ± 2.85	−1.00 ± 0.84	0.0020
	T3	6.02 ± 2.84	−2.01 ± 1.27	6.02 ± 2.75	−1.38 ± 0.93	0.0041
**CK Parameters** ^**a**^						
**Erythema Index**	T0	348.86 ± 76.84		336.93 ± 78.76		
	T1	320.55 ± 56.91	−18.29 ± 14.74	314.44 ± 65.99	−12.03 ± 20.06	0.1763
	T2	309.52 ± 59.39	−29.32 ± 15.2	305.82 ± 66.65	−20.64 ± 25.01	0.0298
	T3	312.78 ± 80.07	−36.09 ± 20.80	311.04 ± 85.44	−25.88 ± 25.86	0.0013
**Skin hydration**	T0	58.59 ± 10.60		59.31 ± 10.32		
	T1	59.30 ± 10.93	1.49 ± 1.58	59.10 ± 10.69	0.68 ± 1.61	0.6283
	T2	60.51 ± 11.46	2.69 ± 2.74	60.56 ± 10.34	2.14 ± 2.69	0.9198
	T3	62.68 ± 10.91	4.87 ± 2.76	61.62 ± 10.12	3.19 ± 2.60	0.0268
**Transepidermal** **Water Loss**	T0	16.01 ± 3.17		15.82 ± 3.20		
	T1	15.85 ± 2.21	−0.43 ± 1.39	15.85 ± 2.30	−0.18 ± 1.42	0.9916
	T2	15.03 ± 2.57	−1.25 ± 1.67	15.71 ± 2.99	−0.32 ± 1.50	0.0701
	T3	14.32 ± 2.28	−1.96 ± 1.88	14.73 ± 2.64	−1.31 ± 2.22	0.1877

^a^ Includes all patients enrolled in the study (*n* = 36). Missing data in the Full Analysis Set (FAS) for primary efficacy endpoints were imputed using the last observation carried forward (LOCF) method.

#### 3.2.2 CK Skin Physiological Function Test

The erythema index decreased progressively on both sides. The reduction did not differ significantly between sides at T1 (−18.29 ± 14.74 vs. −12.03 ± 20.06; *p* = 0.176), but was significantly greater on the combination side at both T2 (*p* = 0.029) and T3 (−36.09 ± 20.80 vs. −25.88 ± 25.86; *p* = 0.0013; Figure 2b).

Regarding skin barrier function, skin hydration increased on both sides continuously. The increase was comparable at T2 (*p* = 0.919) but became significantly greater on the combination side by T3 (*p* = 0.025). Transepidermal water loss (TEWL) values declined on both sides, with no statistically significant difference in the reduction between sides at T3 (*p* = 0.187) (Table 2).

### 3.3 Sensitivity Analysis

After excluding the three dropouts, the IPL + SSA group still showed significantly greater improvements in VISIA and CK values compared to the IPL‐alone side (*p* < 0.05).

### 3.4 Secondary Outcome

#### 3.4.1 Clinical Erythema Assessment

Both the combination side and the IPL‐alone side showed significant improvements in CEA scores at the final assessment (T3) (both *p* < 0.001). No statistically significant difference in the magnitude of improvement was found between the two sides (*p* = 0.500) (Table S1).

#### 3.4.2 Patient Self‐Assessment of Symptoms

At the final assessment (T3), patient‐assessment scores for facial redness (*p* < 0.001), itching (*p* < 0.001), and burning sensation (*p* = 0.004) all improved significantly compared to baseline (Table S2).

#### 3.4.3 Dermoscopy

**FIGURE 3:**
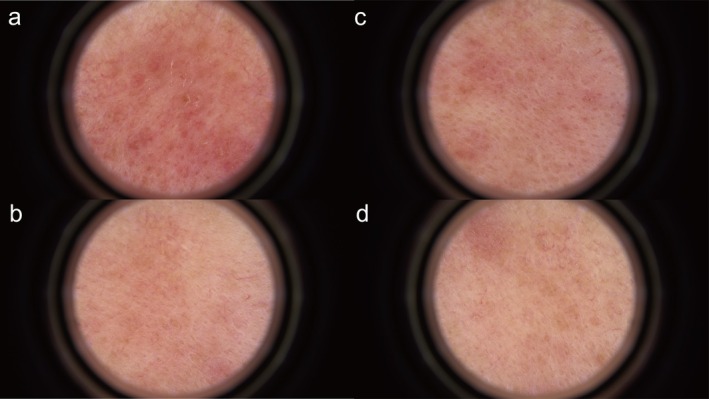
Dermoscopic features of rosacea at baseline and the final follow‐up (Week 12). (a, b) Combination side at baseline (a) and final follow‐up (b), showing a reduction in telangiectasia and background erythema. (c, d) IPL‐alone side at baseline (c) and final follow‐up (d).

At the baseline, dermoscopy revealed diffuse erythema on a light red background, accompanied by polymorphous, tortuous, and dilated capillaries (Figure 3a,c).

After treatment, the combination side showed significant lightening and reduction in the area of background erythema compared to the IPL‐alone side. The number of dilated capillaries decreased, and the remaining vessels became less distinct. These improvements were superior to those observed on the IPL‐alone side. Additionally, follicular ostium dilation improved on the combination side, with reduced or absent keratotic plugs and finer follicular outlines (Figure 3b,d).

#### 3.4.4 Safety Evaluation

All patients experienced expected, transient stinging and erythema during SSA application and immediately post‐IPL, which resolved promptly with cold spray. Post‐treatment adverse events occurred in 6 cases (18.2%) on the combination side (4 erythema, 2 dryness/desquamation) and 3 cases (9.1%) on the IPL‐alone side (3 erythema). There was no statistically significant difference between the two sides (*p* = 0.476). All adverse events were mild, and no serious adverse events were reported.

#### 3.4.5 Satisfaction

Among the 33 respondents, the overall satisfaction rate (combining “satisfied” and “very satisfied” responses) was 72.73% (24/33). Specifically, 17 patients (51.51%) were “satisfied,” and 7 (21.21%) were “very satisfied.” Two patients (6.06%) were “neutral,” and no patient was “dissatisfied” or “very dissatisfied.”

## 4 Discussion

The pathogenesis of rosacea is complex, involving the interplay of multiple factors such as innate immune dysfunction, neurovascular dysregulation, and impaired skin barrier [2]. In this study, we observed that the combination of SSA peel and IPL significantly improved the clinical signs of rosacea. The therapeutic improvement on the combination side was significantly greater than that on the IPL‐alone side, indicating a crucial synergistic role for SSA in the combined treatment.

IPL is an established physical treatment for rosacea. The significant improvement observed on the IPL‐alone side in this study confirms its efficacy. IPL is a non‐coherent, broad‐spectrum light (500–1 200 nm). The 500–600 nm and near‐infrared bands are selectively absorbed by hemoglobin, leading to precise thermocoagulation and occlusion of dilated capillaries, thereby effectively ameliorating facial flushing and persistent erythema [10]. Furthermore, studies indicate that low‐energy IPL can stabilize mast cell membranes and inhibit their activation. This subsequently reduces the release of key inflammatory mediators such as MMP‐9, KLK5, and the cathelicidin LL‐37, alleviating the inflammatory cascade in rosacea [11]. The photobiomodulatory effect of IPL may also stimulate dermal fibroblasts and promote neocollagenesis, which contributes to the repair of skin texture [12].

Salicylic acid has well‐defined anti‐inflammatory properties. It inhibits the activation of the NF‐κB signaling pathway and downregulates the expression of inflammatory cytokines such as IL‐1, IL‐6, and TNF‐α [13]. Studies have confirmed that inhibition of the NF‐κB/NLRP3 axis can significantly restore the expression of tight junction proteins such as claudin‐1, thereby effectively repairing the skin barrier [14]. In addition, salicylic acid is lipophilic, enabling it to penetrate deeply into hair follicles, dissolve excessive keratin plugs, and relieve obstruction of the pilosebaceous duct [15].

Due to its unique “supramolecular” technology, SSA differs from traditional salicylic acid preparations that rely on alcohol solvents and are highly irritating, offering better local tolerability. Meanwhile, this system features temperature‐sensitive sustained release: It is liquid at room temperature and transforms into a semi‐solid hydrogel film upon contact with the skin, which enhances the absorption of active ingredients while significantly reducing irritation to the skin barrier [16, 17]. For patients with rosacea, who commonly have impaired skin barrier function and sensitive skin, this mild, long‐acting, sustained‐release property offers significant advantages. Additionally, unlike acid preparations such as alpha‐hydroxy acids, SSA does not require neutralization with alkaline solutions, providing higher operational safety and enabling favorable synergistic effects with the photothermal action of IPL.

Regarding safety, the combination treatment did not increase the overall incidence of adverse events compared to IPL monotherapy. The transient stinging and erythema associated with SSA were expected and manageable. All patients tolerated the procedures well. However, dryness and desquamation were observed in a few cases, which indicates the necessity of post‐procedure moisturization. To ensure patient comfort, the SSA contact time should be personalized based on the skin's sensitivity, followed by a strict repair routine to protect the already compromised skin barrier.

Currently, comparative studies on laser combined with different acids for the treatment of rosacea are limited. Two studies from China reported the efficacy of IPL combined with azelaic acid, primarily using subjective clinical scoring as the outcome measure [18, 19]. Despite heterogeneity in the evaluation systems, our study is consistent with the above studies on the core conclusion that laser combined with acid therapy can improve rosacea symptoms. This study used objective quantitative measurements as the primary indicators, including the red area measured by the VISIA Complexion Analysis System and the erythema index measured by the CK device, providing a quantitative assessment of clinical efficacy. These parameters offer reproducible objective evaluation metrics for future comparisons among different rosacea treatment regimens. In addition, we supplemented these with the CEA scale and patient self‐assessment of symptoms, establishing a comprehensive and complete evaluation system. In the future, we expect to further explore the effects of different acid types combined with laser therapy using the unified evaluation system.

This study has several limitations. It was a single‐center study with a limited sample size. Furthermore, the follow‐up was relatively short, so the long‐term effect of this combined therapy on the disease cannot be evaluated. Moreover, the study primarily relied on clinical and skin physiological parameters. Future research could incorporate molecular biological analyses to provide more direct laboratory evidence for the synergistic mechanism between SSA and IPL.

## 5 Conclusion

The combination of supramolecular salicylic acid (SSA) peeling and intense pulsed light (IPL) is superior to IPL monotherapy for the treatment of rosacea. This combined modality represents a promising therapeutic strategy with a favorable safety profile.

## Author Contributions

M.W. and A.T. were responsible for all data analysis, tables, and figures, as well as writing the main manuscript text. F.Z. offered good guidance about the constructive discussions. All authors have read and agreed to the published version of the manuscript.

## Funding

This work was supported by the National Natural Science Foundation of China (Grant No. 82273555) and the Beijing Municipal Natural Science Foundation (Grant No. L234068).

## Ethics Statement

This study was prospectively registered in the Chinese Clinical Trial Registry (ChiCTR2500096212). The protocol was approved by the Ethics Review Committee of Beijing Friendship Hospital (Approval No. 2023‐P2‐083‐02).

## Consent

Informed consent was obtained from the patients for the publication of their images.

## Conflicts of Interest

The authors declare no conflicts of interest.

## Supporting information


**Table S1:** Clinical Erythema Assessment (CEA) *n* (%).
**Table S2:** Patient Self‐Assessment of symptoms *n* (%).

## Data Availability

The data that support the findings of this study are available on request from the corresponding author. The data are not publicly available due to privacy or ethical restrictions.
