# Supplementary material for: Efficacy and Safety of Supramolecular Salicylic Acid Combined With Intense Pulsed Light for Rosacea: A Split‐Face Trial
Source: J Cosmet Dermatol. 2026 Jun 22;25(7):e71007. doi: 10.1111/jocd.71007 (PMC13287546; doi:10.1111/jocd.71007)
Supplement: Supplementary file 1 — Table S1: Clinical Erythema Assessment (CEA) n (%). Table S2: Patient Self‐Assessment of symptoms n (%). [file JOCD-25-e71007-s001.docx]

**Table S1** Clinical Erythema Assessment (CEA) **n(%)**

| **Time** | **CEA score** | **Combination Side** | **IPL-alone side** |
| --- | --- | --- | --- |
| **T0** |  |  |  |
|  | 1 | 0 | 0 |
|  | 2 | 15(45.45) | 16(48.48) |
|  | 3 | 12(36.36) | 11(33.33) |
|  | 4 | 6(18.18) | 6(18.18) |
| **T3** |  |  |  |
|  | 1 | 11(33.33) | 10(30.30) |
|  | 2 | 18(54.55) | 18(54.55) |
|  | 3 | 2(6.06) | 3(9.09) |
|  | 4 | 2(6.06) | 2(6.06) |
| **P value** |  | <0.001 | <0.001 |

^a^ Includes patients who completed the study(n=33)

**Table S2** Patient Self-Assessment of symptoms **n (%)**

|  | **Redness** | **Pruritus** | **Burning** |
| --- | --- | --- | --- |
| **Grade** |  |  |  |
| **I** | 9(27.27) | 14(42.42) | 24(72.73) |
| **II** | 17(51.52) | 13(39.39) | 7(21.21) |
| **III** | 7(21.21) | 6(18.18) | 2(6.06) |
| **IV** | 0 | 0 | 0 |
| **P value** | <0.001 | <0.001 | 0.004 |

^a^ Includes patients who completed the study(n=33)
